# Factors influencing net ecosystem carbon change in cold-temperate coniferous forests of the Da Xing’an Mountains: analysis across developmental stages based on stand, structural, and environmental factors

**DOI:** 10.3389/fpls.2025.1663271

**Published:** 2025-11-24

**Authors:** Zirui Wang, Yuanshuo Hao, Lihu Dong, Zheng Miao, Xingji Jin, Xuehan Zhao, Shoumin Cheng

**Affiliations:** 1 College of Forestry, Northeast Forestry University, Harbin, Heilongjiang, China; 2 Key Laboratory of Sustainable Forest Ecosystem Management-Ministry of Education, School of Forestry, Northeast Forestry University, Harbin, Heilongjiang, China

**Keywords:** net ecosystem carbon change, environmental factors, stand age, natural forests, the Da Xing’an Mountains

## Abstract

**Introduction:**

The Da Xing’an Mountains region harbors China's only cold-temperate coniferous forests and serves as a critical ecological barrier, playing a vital role in forest ecosystems and carbon sequestration. Stand age, shaped by population dynamics, disturbance regimes, and management practices, significantly influences the global carbon cycle. Although forest development is known to correlate with productivity shifts, how production varies across specific stand developmental stages and the relative contributions of driving factors remain poorly understood.

**Methods:**

Using data from the National Forest Continuous Inventory (NFCI, 2005–2010) in the eastern Da Xing’an Mountains, we analyzed the effects of stand characteristics, structural diversity, and environmental variables on Net Ecosystem Carbon Change (NECC) across a spectrum of developmental stages, from young to overaged forests.

**Results:**

Our findings demonstrate that: (1) Net Ecosystem Carbon Change (NECC) is co-limited by stand characteristics, structural diversity, and environmental factors, with stand characteristics exerting the strongest influence, primarily via direct effects. (2) As stands develop, the impacts of structural diversity (effect increasing from 8.68% to 16.44%) and soil factors (from 8.80% to 10.30%) on productivity intensify. (3) In contrast, the influence of climate (decreasing from 30.40% to 17.67%) and terrain (from 14.55% to 6.28%) diminishes with advancing growth stages.

**Discussion:**

This study provides a comprehensive, system-level analysis of the determinants of Net Ecosystem Carbon Change (NECC). By integrating multiple drivers, our work establishes a theoretical foundation for predicting Net Ecosystem Carbon Change (NECC) changes under global change scenarios. These insights are crucial for formulating effective forest management strategies to mitigate the challenges of climate change and biodiversity loss.

## 1 Introduction

Net Ecosystem Carbon Change (NECC) is a key component of carbon fluxes in terrestrial ecosystems and plays a vital role in advancing the understanding of global carbon cycles and land–atmosphere interactions (Shiri et al., 2022). NECC serves as an important metric for assessing the balance of the carbon cycle and is also a critical indicator for evaluating the effects of meteorological variations on ecosystem carbon balance. Therefore, clarifying the factors influencing NECC is essential for scientifically assessing the carbon source/sink capacity of a region. Many studies have evaluated NECC (Shuai et al., 2019; Zhang et al., 2024a). While this static perspective of NECC offers essential information on the present conditions of forest carbon storage, it does not adequately clarify the dynamic temporal changes within forest ecosystems and their responses to environmental variations. NECC is influenced by three processes: the growth of mature trees, the recruitment of new individuals, and carbon loss resulting from mortality (Yuan et al., 2019). For a long time, species diversity (Barrufol et al., 2013), structural diversity (Wang et al., 2021), climate (Jing et al., 2022), and stand characteristics (Lin et al., 2021) have been considered the primary determinants of NECC. However, in forests, the composition of tree species frequently changes as the forest ages, which is a process that might take several decades. During this period, the influence of environmental factors on ecosystem functioning may be altered (Zhao et al., 2024). Understanding the variation in NECC between phases of forest development, from the initial phase of secondary succession to old growth, is essential for restoring ecosystem functionality in damaged environments.

The prevalent view is that environmental elements in forests, including climate and site conditions, along with stand structure, significantly influence NECC (Du et al., 2022; Zhang et al., 2024a). Climate elements are regarded as the principal environmental determinants influencing NECC at a regional scale (Sun et al., 2021), with temperature and precipitation serving as the fundamental drivers of spatiotemporal patterns of NECC (Xi and Yuan, 2022). However, at smaller spatial scales, factors such as stand structure, terrain, and soil significantly influence the spatial variability of NECC (Jian et al., 2022). Furthermore, researchers have reported that in temperate larch forests, the beneficial impact of rising temperatures on the wood supply decreases as the forest matures. Forest production progressively increases, stabilizes, and subsequently decreases as the forest develops (Kira and Shidei, 1967). In northern and mountainous areas, elevated temperatures increase arboreal development and prolong the growing season of vegetation. Under climate change conditions, forests are expected to exhibit accelerated growth, reach maturity sooner, and experience earlier mortality, resulting in varied responses to climate change among forests of different ages (Collalti et al., 2019). Conversely, studies have determined that stand structure is positively connected with NECC (Wang et al., 2021), suggesting that within a stand, trees enhance NECC through resource allocation, facilitation, and biological feedback. Nonetheless, the significance of stand structure in forecasting NECC may evolve over time, as tree interactions intensify with growth during forest succession (Wang et al., 2022). Consequently, it is imperative to account for variations at various ages when forecasting the impacts of environmental conditions and stand structure on NECC.

Moreover, stand variables, including age and density, are crucial determinants of NECC. Stand density indicates the area used by trees and their internal horizontal structure, which affects the forms of tree development, NECC, and ecological stability (Liu et al., 2022). Increased stand densities enhance forest carbon storage and timber output by increasing canopy density, thereby increasing the amount of light captured. Stand age is a significant determinant of biomass and NECC (Liu et al., 2018). Stand age can increase biomass and NECC through increases in tree size (Becknell and Powers, 2014) and variations in size (Zhang and Chen, 2015). Throughout the forest restoration process, tree size, stand age, stand density, and species composition substantially change, whereas NECC markedly increases. Nonetheless, the primary biological processes influencing NECC remain a debated subject.

In recent years, machine learning technology has demonstrated superiority over traditional statistical methods by overcoming the limitations of big data analysis (Christin et al., 2018; Özçelik et al., 2013) and is regarded as a potent tool that is extensively utilized in forestry (Jevšenak and Skudnik, 2021; Lan et al., 2023; Wu et al., 2019). The random forest (RF) algorithm has been widely employed to assess variable significance and facilitate variable selection because of its ability to effectively mitigate overfitting and variation (Lin et al., 2004). Numerous studies have employed linear statistical approaches to examine the correlations between NECC and numerous variables, frequently overlooking the direct or indirect effects of these factors. Furthermore, identifying the ideal range of environmental variables using these methods requires substantial sample sizes and may not adequately elucidate the real mechanisms driving variations in NECC due to stand and environmental factors (Zhang et al., 2024a). Partial least squares structural equation modeling (PLS–SEM) is extensively employed to analyze the direct, indirect, and cumulative impacts of specific variables on other variables. This approach accommodates nonnormal distributions, nonlinear associations, and scenarios with numerous variables (Hair et al., 2021). Integrating the RF model with PLS–SEM to investigate the physiological principles of NECC may enhance the understanding of forest dynamics and serve as a reference in the context of impending climate change.

The Da Xing’an Mountains, situated in Northeast China, form a delicate mountainous region with abundant resources (He et al., 2021). This abundant-resource region maintains an unspoiled cold-temperate coniferous forests ecology, being China’s sole boreal coniferous forest area and one of the few extant biological gene pools (Zhao et al., 2024). The forests of the Da Xing’an Mountains are essential for ecological security in northeastern China and the broader North China region, and their function in the carbon cycle is significant (Jiang et al., 2002). This location has garnered considerable interest from forest managers in recent research (Wang et al., 2008). Nonetheless, prolonged human logging activities have significantly compromised stand structure, functionality, and stability, hence substantially impeding the sustainable growth of the forest ecosystem in the Da Xing’an Mountains (Wu et al., 2022). Consequently, statistically assessing the impacts of stand structure and environmental variables on NECC and providing scientifically valid restoration strategies are imperative. This assessment is crucial for reinstating biological functions, increasing forest quality, and increasing the capacities of carbon sequestration and sinks in the Da Xing’an Mountains.

**Figure 1:**
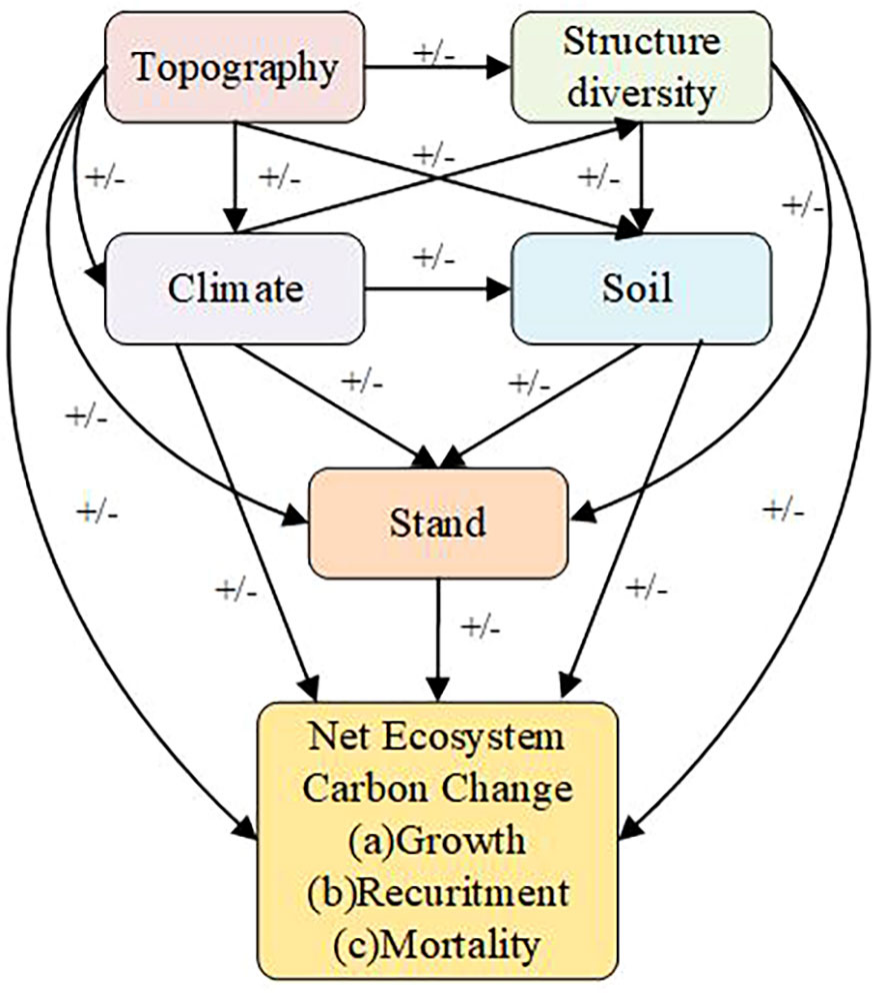
A conceptual model revealing the expected links of environmental factors (topography, climate, and soil nutrients), structure, and stand factors on net ecosystem carbon change demographic processes (recruitment, growth, and mortality). Hypothesized positive, negative, and unknown effects are indicated by +/- signs.

In this research, 782 natural forest plots were evaluated, and RF and PLS–SEM were utilized to ascertain the direct and indirect influences of stand characteristics, structural diversity, and environmental variables on NECC. Our dataset included forests at various developmental stages, from the initial phases of secondary succession to mature old-growth forests. We categorized these plots into five forest developmental stages on the basis of stand age to investigate the variations in production as the forests matured. Consequently, we propose ways to increase Net Ecosystem Carbon Change in the Da Xing’an Mountains to improve forest quality. The primary aims of this study are as follows:(1) How do stand characteristics, structural diversity, and environmental variables collectively influence NECC in the cold-temperate coniferous forests of the Da Xing’an Mountains? (2) What are the relative impacts and evolutionary trends of stand characteristics, structural diversity, and environmental variables on NECC across different stages of forest development? For these objectives we use the conceptual model outlined in Yuan et al. as a starting point(**Figure 1**), we tested the following hypotheses: (1)All variables had direct effects on growth, recruitment and mortality, and indirect effects on net ecosystem carbon change. (2)Topography affected climate, structure diversity, soil, stand. (3)Climate affected structure diversity, soil, stand. (3)Structure diversity affected soil, stand. (4)soil affected stand.

## 2 Materials and methods

### 2.1 Study area and sample plot data

**Figure 2:**
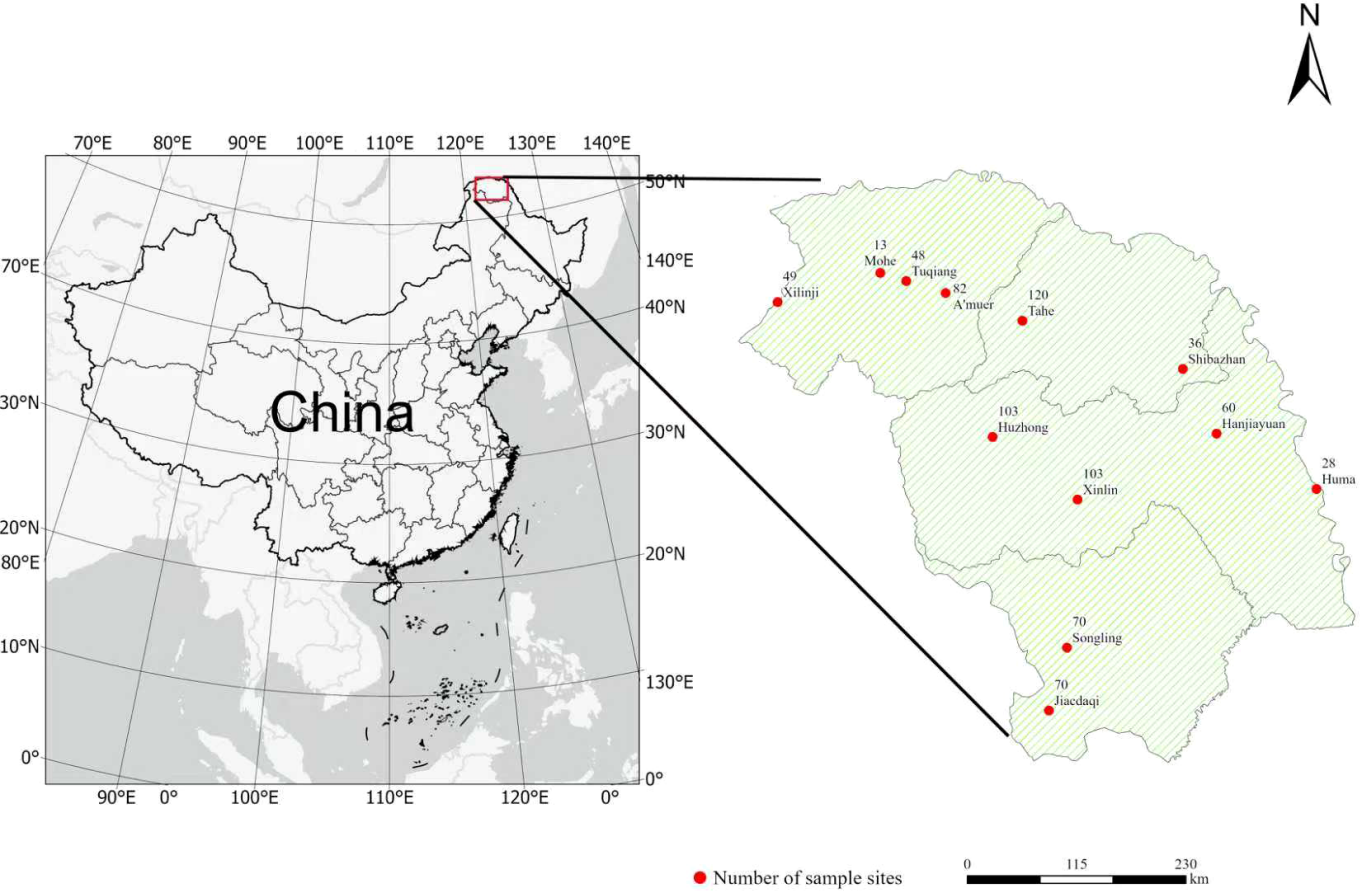
Geographical locations of the study area in the eastern Da Xing’an Mountains, Northeast China.

The research area is located in the key state-owned forest region of the Da Xing’an Mountains in Heilongjiang Province. Geographically, the area is located in northwestern Heilongjiang Province, northeastern Inner Mongolia Autonomous Region, and on the northeastern slope of Da Xing’an ridge (**Figure 2**). It spans 121°10’53”-127°01’21”E, 50°07’02”-53°33’42” N and encompasses a total area of 8.02 × 10^6^ hectares. The area has a distinct cold–temperate continental monsoon climate, with an annual average temperature of -2 °C. Annual precipitation values range from 430–460 mm, and precipitation is concentrated from July–September. The primary soil types in the area include brown coniferous forest soil, dark brown soil, gray–black soil, meadow soil, and marsh soil. The climax community of the forest ecosystem in the Da Xing’an Mountains comprises a bright coniferous forest typical of a cold–temperate zone. The dominant tree species include *Larix gmelinii*, *Betula platyphylla*, *Populus davidiana*, *Quercus mongolica*, and *Pinus sylvestris* var. *mongolica*, among others.

The data for this study were sourced from the seventh and eighth national forest resource inventories (NFCIs) of the Da Xing’an Mountains. Utilizing systematic sampling and a kilometer grid approach, 782 sample plots were set up over a span of five years. In the Da Xing’an Mountains, the fixed sample areas were organized in a grid measuring 8 km by 8 km, each spanning 0.06 hectares. Measurements were taken for various tree species, breast height diameters (≥ 5 cm), and the mean age of the stands in every fixed plot.

### 2.2 Calculating carbon sequestration in the forest stands of permanent sample plots

The biomass of each tree was obtained by substituting the tree measurement data into a biomass model. Subsequently, the biomass was multiplied by the ratio of the carbon content of each species to ascertain the carbon storage capacity of every tree. The term ‘growth’ denotes the variation in the carbon sequestration of preserved trees across two survey intervals; ‘recruitment’ indicates the rise in carbon sequestration due to trees having a diameter at breast height (DBH) < 5 cm in the initial survey, increasing to 5 cm in the subsequent survey; ‘mortality’ denotes the reduction in carbon storage resulting from tree death during the survey. This research defines NECC as the aggregate of growth, mortality, and recruitment.

### 2.3 Stand variables

The variables in our study consisted of stand age, stand density, and DBH. The stand age was ascertained by computing the arithmetic mean of the ages of the typical dominant trees via tree ring analysis. The DBH for each plot was computed for all trees within the plot. The stand density (N·ha^−1^) was calculated by dividing the total tree count in the plot by the plot area.

### 2.4 Structural diversity indicators

We employed the diversity index of the tree species composition to delineate structural diversity. This index was utilized to evaluate data, including the quantity of tree species, their relative abundance, and the fraction of species biomass. It also accurately depicts the uniformity and extent of species intermingling within a stand.

### 2.5 Environmental data

Climate data were obtained from the ClimateAP (v2.30) application, which generates climate variables on the basis of latitude, longitude, and elevation of the sample plots. We obtained soil data from the Harmonized World Soil Database (HWSD) of the Food and Agriculture Organization (http://www.fao.org/faostat/en/#data) (Milovac et al., 2018), which offers extensive soil property and nutrient information at a resolution of 1000 m. Using kriging interpolation, the data were resampled to a 30 m resolution and extractable by the geographic coordinates of a sample plot. Topographic data were acquired during the creation of sample plots, and a GPS was used to identify and document topographic information accurately. In total, there were 16 climate factors, 10 soil variables, and 3 topographic variables, as shown in **Table 1**.

**Table 1:** Description of the environmental data used in this study.

Factors	Variables	Units	Description
Climate	AHM	_	Annual heat: moisture index
	CMD	mm	Hargreaves climatic moisture deficit
	DD_0	Days	Degree-days below 0 °C
	TD	°C	Temperature difference between MWMT and MCMT, or continentality
	DD18	days	Degree-days above 18 °C
	DD5	days	Degree-days above 5 °C
	EMT	°C	Extreme minimum temperature over a 30- year period
	EXT	_	Extreme maximum temperature over a 30- year period
	EREF	Mm	Hargreaves reference evaporation
	MAP	mm	Mean annual precipitation
	MAT	°C	Mean annual temperature
	MCMT	°C	Mean coldest month temperature
	MWMT	°C	Mean warmest month temperature
	NFFD	Days	Number of frost-free days
	PAS	Mm	Precipitation as snow between August in previous year and July in current year
	RH	%	Relative humidity
Soil	DEPTH	cm	Reference soil depth
	REF_BULK_DENSITY	kg/dm^3^	Soil reference bulk density
	OC	weight %	Soil organic carbon
	PH_H_2_O	-log(H^+^)	Soil pH (H_2_O)
	CEC_SOIL	cmol/kg	Soil CEC (soil)
	BS	%	Soil base saturation
	TEB	cmol/kg	Soil TEB
	CACO_3_	weight %	Soil calcium carbonate
	ESP	%	Soil sodicity (ESP)
	ECE	dS/m	Soil salinity (ECE)

### 2.6 Statistical analyses

Pearson correlation analysis was used to initially assess the significant association of prospective predictors associated with NECC (at the 0.05 level) and the threshold for the correlation coefficient between predictor variables and NECC. The predictors were utilised for subsequent analysis only if the absolute values of their correlation coefficients above 0.2 (threshold of 1). Second, the absolute value of the correlation coefficient among the significant candidate variables themselves was guaranteed to be < 0.4 (threshold 1). A predictor was excluded if the absolute value of the correlation coefficient between itself and all other predictors was greater than 0.4, and the variance inflation factor (VIF) was used in stepwise regression analysis to evaluate the multicollinearity. All the VIF values < 10 indicate that collinearity between variables has no significant impact on our results (Graham, 2003). The VIF and Pearson methods were implemented in SPSS software (version 23.0).

The RF algorithm chooses the variables on the basis of the importance scores of the input variables. In the RF algorithm, the importance value was calculated by permuting on out-of-bag (OOB) data: (1) the prediction error (the mean sum of the squares of residuals, MSE) on the OOB portion of the data was recorded for each tree, (2) the same was done after permuting each predictor variable, and (3) the difference between the two was then averaged over all trees as importance scores (Grömping, 2009). The importance scores of all the predictors were normalized to a percentage. The RF method was implemented in the randomForests package in the R platform (version 4.3.2).

Before statistical analysis was conducted, the classical theory of ecology was used to divide the life cycle of a forest into five developmental stages: young forest, middle-aged forest, near-aged forest, mature forest, and overaged forest. During various stages, changes in growth rates, biomass buildup and photosynthetic efficiency result in different degrees of NECC. Consequently, tree species and age were categorized into separate classifications for analysis. Consequently, we categorized the trees on the basis of various tree types and stand ages. *Larix gmelinii*, *Pinus sylvestris*, and *Picea asperata* were categorized on the basis of the following age ranges: ≤ 40, 41–60, 61–80, 81–120 and > 120 a. *Populus davidiana* and *Betula platyphylla* were categorized into the following age groups: ≤ 30, 31–50, 51–60, 61–80 and > 80 a. The age classification for *Quercus mongolica* and *Betula davurica* was as follows: ≤ 40, 41–60, 61–80, 81–120 and > 120 a. PLS–SEM was employed to investigate the direct, indirect, and interactive links among different variables affecting the response ratio (RR) of agricultural yields. The net effect of one variable on another was determined by integrating all direct and indirect pathways connecting the two variables. The route coefficients and coefficients of determination (R²) were computed using the R package “plspm”. All data analysis was conducted using R version 4.0.2.

## 3 Results

### 3.1 Changes in net ecosystem carbon change traits with forest development

The mean carbon reserves in this region’s natural forests stood at 33.35 t·hm^-2^ in 2010, which was a 4.38 t·hm^-2^ increase from 2005. On average, NECC was 1.55 t·hm^-2^·a-1, accompanied by growth, recruitment, and mortality rates of 0.88 t·hm^-2^·a^-1^, 0.23 t·hm^-2^·a^-1^, and 0.43 t·hm^-2^·a^-1^, respectively. Generally, as forests aged, there was a notable reduction in both growth rates and entry rates, alongside an increase in mortality rates. The mortality percentages for forests categorized as young, middle-aged, near-mature, mature, and overaged were 6.73%, 24.66%, 19.28%, 23.77%, and 30.49% of the overall mortality, respectively (**Table 2**).

**Table 2:** Statistics of the basic characteristics of the sample plots.

Age group	Initial carbon stock /(t·hm^-2^)	Ending carbon stock /(t·hm^-2^)	Recruitment/(t·hm^-2^·a^-1^)	Growth/(t·hm^-2^·a^-1^)	Mortality/(t·hm^-2^·a^-1^)	Net Ecosystem Carbon Change /(t·hm^-2^·a^-1)^
Young stand	11.23 ± 13.48a	17.38 ± 14.44a	0.57 ± 0.43b	1.23 ± 0.79d	0.15 ± 0.39a	1.94 ± 1.00c
Middle-aged stand	35.02 ± 20.44b	39.77 ± 20.27b	0.16 ± 0.18a	0.94 ± 1.04c	0.55 ± 0.77ab	1.66 ± 0.68b
Near-mature stand	39.67 ± 18.39bc	43.33 ± 18.49bc	0.11 ± 0.14a	0.79 ± 0.78bc	0.43 ± 0.67b	1.33 ± 0.60a
Mature stand	43.75 ± 20.64c	46.96 ± 20.69c	0.13 ± 0.17a	0.65 ± 0.87b	0.53 ± 0.69ab	1.30 ± 0.66a
Overmature stand	50.85 ± 23.50d	52.64 ± 22.96d	0.13 ± 0.20a	0.36 ± 0.94a	0.68 ± 0.87c	1.17 ± 0.69a
Total	33.35 ± 22.85	37.73 ± 22.28	0.23 ± 0.31	0.88 ± 0.92	0.43 ± 0.69	1.55 ± 0.80

Data with the same small letter in each panel indicate no significant difference at the P = 0.05 level (Duncan test).

### 3.2 Significant variables affecting stand biomass via multiple feature selection methods

In the examination of NECC, considerable discrepancies were observed in the number of variables chosen by the three feature selection approaches, and the selected variables varied among these methods. For example, relative humidity (RH) values were identified as significant using correlation analysis and redundancy analysis but excluded by alternative methods. Moreover, many feature selection strategies exhibited limited consistency in terms of the chosen variables. Age, Dg and density in stand structural diversity were consistently significant variables across all three techniques. Ultimately, five critical biodiversity factors were identified by the intersection approach, as follows: (1) climate: MAT and MAP; (2) stand: age, mean diameter at breast height (DBH, Dg), and density; (3) soil: depth; (4) structural diversity: ISCD; and (5) topography: altitude.

### 3.3 Results of partial least squares path modeling

**Figure 3:**
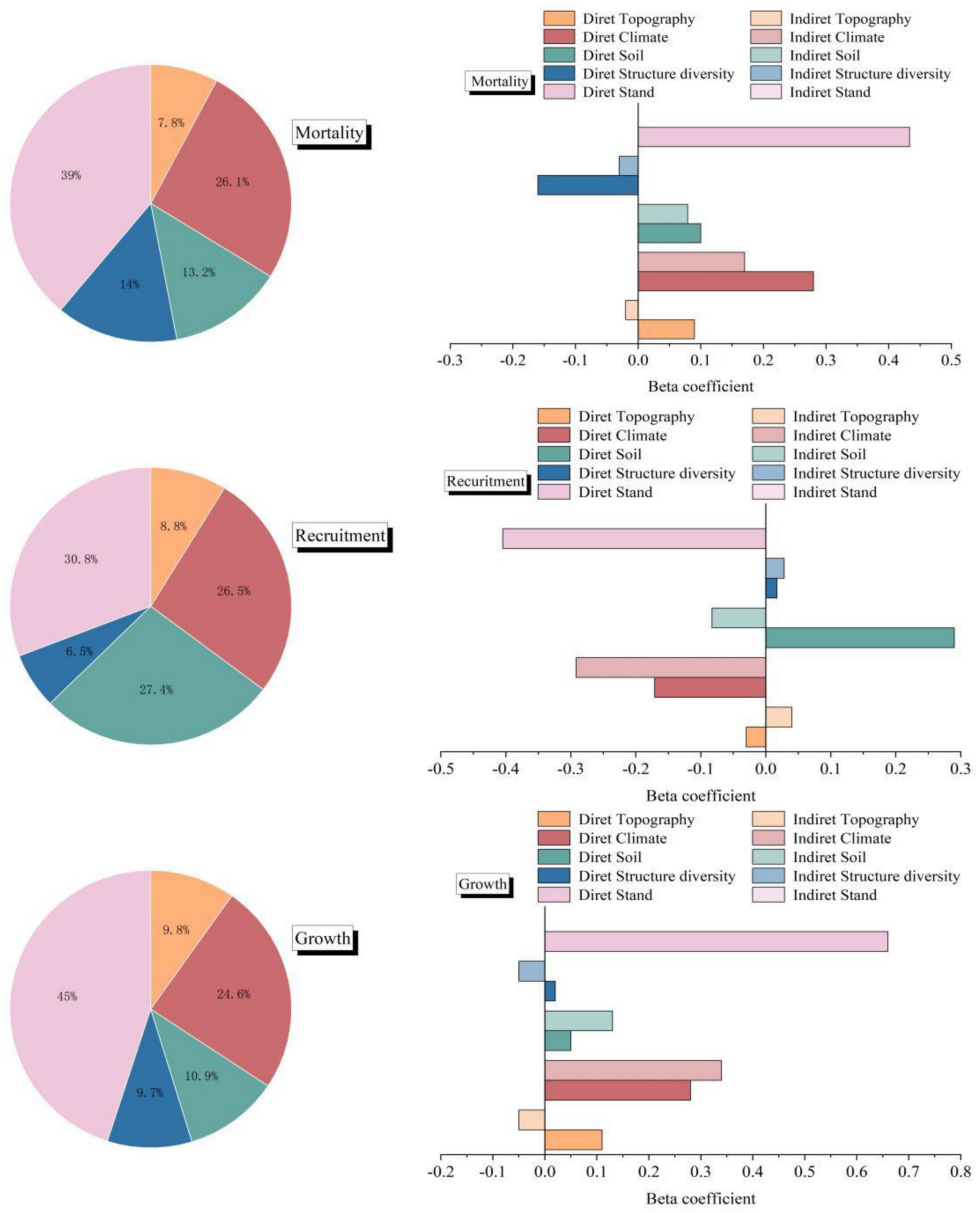
Beta coefficients and the relative contributions of stand factors, structural diversity, and environmental variables to net ecosystem carbon change. The filled bars indicate direct effects, and the striped bars indicate indirect effects of stand factors, structural diversity, and environmental variables on growth, recruitment, and mortality. The pie charts show the relative importance of each predictor for growth, recruitment, and mortality.

The variables identified by the VIF, Pearson, and RF methods were incorporated into the PLS–SEM with NECC (growth, recruitment, and mortality). The PLS–SEM performed well, with a strong explanatory ability for the causal paths. Specifically, the AVE, alpha, CR, and Rho_A values indicated that the model’s fit was within an acceptable range. The goodness-of-fit (GOF) values revealed that the model’s overall quality was quite high (**Table 3**). The analysis indicated that stand factors, structural diversity, and environmental variables contributed 68.2%, 39.2%, and 35.2%, respectively, to the variability in growth, recruitment, and mortality. Furthermore, growth, recruitment, and mortality jointly accounted for 93.8% of the variation in NECC, underscoring their pivotal role in influencing NECC dynamics (**Figure 3**).

**Table 3:** Model performance of the PLS–SEM.

Types	GOF	AVE	Cronbach’ alpha	CR	Rho_A
Topography	/	1.00	1.00	1.00	1.00
Climate	/	0.78	0.64	0.80	0.73
Soil	/	1.00	1.00	1.00	1.00
Structural diversity	/	1.00	1.00	1.00	1.00
Forest	/	0.99	0.99	1.00	0.99
Values	0.54	/	/	/	/

**Figure 4:**
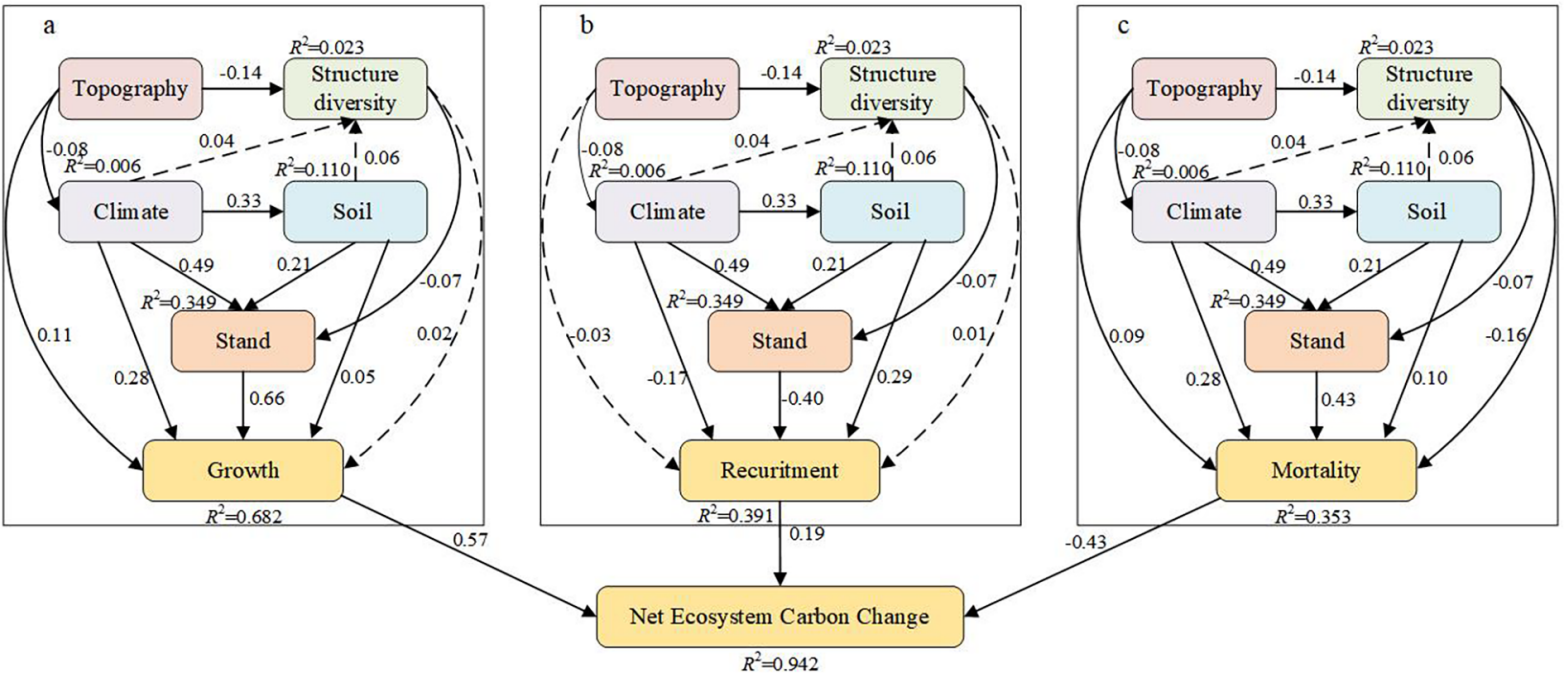
PLS–SEM revealed the effects of stand factors, structural diversity, and environmental variables on Net Ecosystem Carbon Change The climate variables included MAT and MAP; the stand attributes included DBH, stand density, and stand age; the topographic factor was elevation; the soil property was soil depth; and the structural diversity was represented by the diversity index of the tree species composition. **(A)** The effects of stand factors, structural diversity and environmental factors on Growth. **(B)** The effects of stand factors, structural diversity and environmental factors on Recuritment. **(C)** The effects of stand factors, structural diversity and environmental factors on Mortality. The solid and dotted lines indicate that the path is significant (P<0.05) and insignificant (P>0.05), respectively.

The growth of a stand was directly influenced by factors such as stand (b=0.66), climate (b=0.28), topography (b=0.05), structural diversity (b=0.02), and soil (b=0.08; **Figure 4a**). The increase in stand growth was particularly evident due to stand features, as optimal tree density fosters stand efficacy, facilitates canopy shading, and improves water-use efficiency. Nonetheless, overall, topography constrained stand growth, as elevated altitudes typically result in lower temperatures, which is a critical determinant of plant growth, hence substantially impeding vegetation survival and development. The advantageous influence of climatic circumstances on NECC was significant, particularly as regions with elevated temperatures and more precipitation typically exhibit enhanced forest growth (**Figure 4a**).

The PLS–SEM analysis for recruitment indicated that stand factors exerted the most substantial positive direct influence on recruitment (b = -0.40). Structural variety and soil were positively correlated with recruitment (b=0.02, b=0.21), but climate had a negative impact on recruitment (b=-0.17; **Figure 4b**). Although topography did not exert a substantial direct influence on recruitment, both factors indirectly affected recruitment because of their negative correlation with climate and positive association with stands (**Figure 4b**).

The PLS–SEM analysis of mortality revealed the indirect impacts of topography (b = 0.10), climate (b = 0.28), structural variety (b = -0.19), and soil (b = 0.19) on mortality via their interactions with stands (b = 0.43; **Figure 4c**). In the statistical examination of the three separate carbon pools, growth and death constituted the primary sources of variation in NECC, with recruitment following thereafter (**Figure 4c**).

### 3.4 Factors controlling net ecosystem carbon change across different stand ages

**Figure 5:**
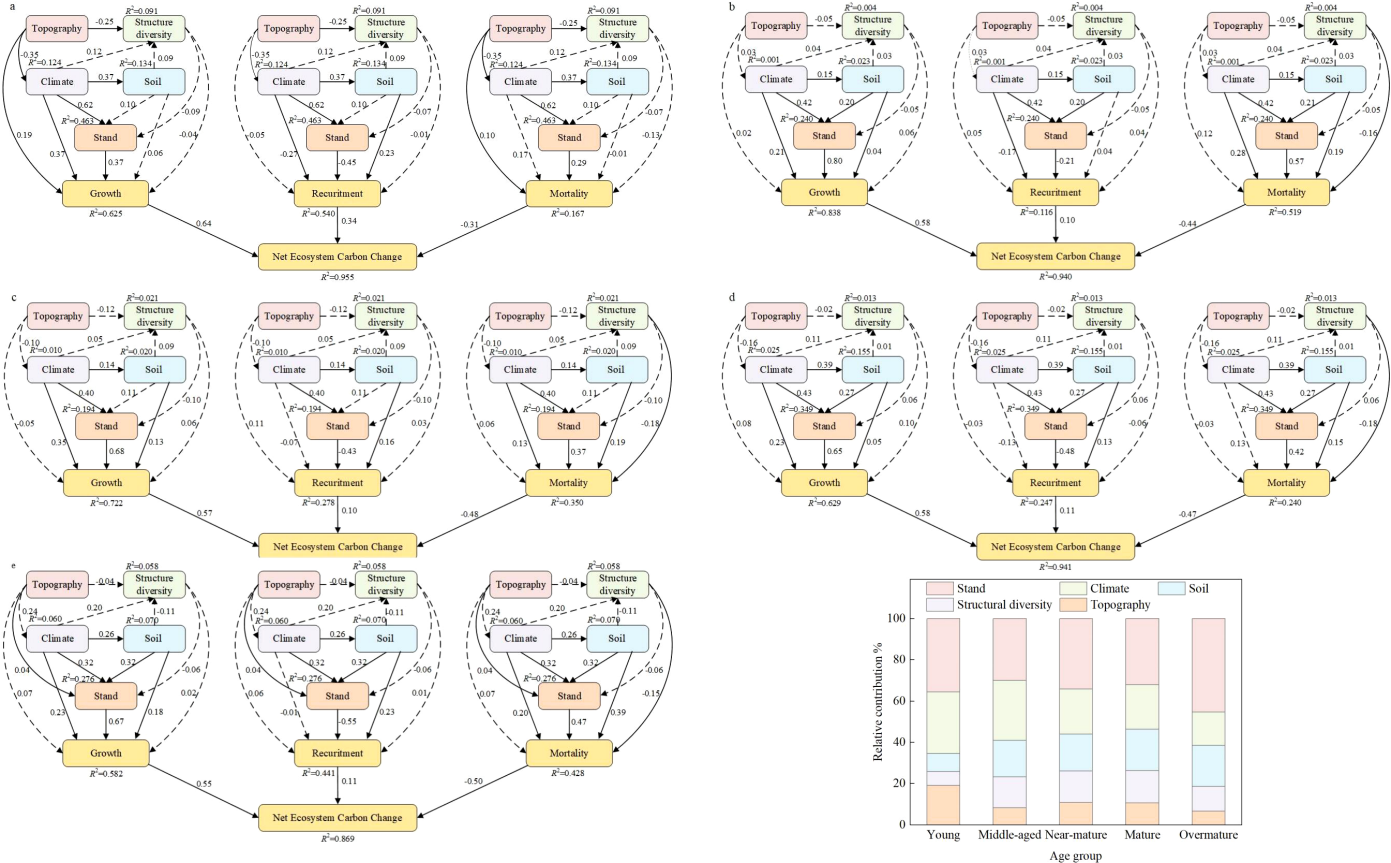
PLS-SEM relating net ecosystem carbon change to the influencing factors at the five development stages. **(a)** young stand, **(b)** Middle-ages stand, c near-mature stand, **(d)** mature stand, **(e)** Over-mature stand. **(f)** The relative contribution of the influencing factors on net ecosystem carbon change based on variance partitioning analysis at the five development stages.

We constructed a PLS-SEM to examine the linkages among topography, climate, structural diversity, stand characteristics, soil properties, and NECC. Our analysis revealed significant direct and indirect effects of topography, climate, structural diversity, stand characteristics, and soil on NECC. PLS-SEM analyses were also conducted separately for five distinct stand development stages. We confirmed that the influence of topography on NECC decreased with stand development. The direct effects of topography on growth and mortality were strongest and statistically significant in the seedling stand stage (**Figure 5a**). These direct effects diminished and became non-significant in subsequent development stages. Similarly, the influence of climate on NECC declined with stand development. The direct effects of climate on growth and recruitment peaked during the seedling stage (**Figure 5a**). Notably, climate’s effect on recruitment became non-significant in the immature stand stage (**Figure 5b**). Unexpectedly, climate’s effect on mortality exhibited a strengthening trend as stands developed. Conversely, the influences of soil and structural diversity on NECC increased with stand development. The direct effect of soil on NECC reached its maximum in the mature (**Figure 5d**) and over-mature stand stages (**Figure 5e**). Structural diversity’s effect on growth and mortality shifted from negative in the seedling stage (**Figure 5a**) to positive in the immature stand stage (**Figure 5b**).

Across the five development stages, the influence of climate (from 30.40% to 17.67%) and topography (from 14.55% to 6.28%) on NECC decreased with stand development. Conversely, the influence of structural diversity (from 8.68% to 16.44%) and soil (from 8.80% to 10.30%) increased across the stand development stages (**Figure 5f**). However, the effect of structural diversity on NECC was most pronounced in the sub-mature stand stage, while the effect of soil properties was most pronounced in the immature stand stage. Furthermore, as stands approached the mature/over-mature stage, the relative importance of structural diversity to NECC exceeded that of the environmental variables (**Figure 5f**).

## 4 Discussion

### 4.1 Impact of stand factors and structural diversity on net ecosystem carbon change

We employed PLS–SEM to analyze the multivariate links driving stand factors and structural diversity. The PLS–SEM effectively elucidated the relationships utilizing the variables chosen through the intersection strategy. The stand variables (age, dg, and density) significantly contributed to net ecosystem carbon change. Researchers have demonstrated that stand density directly influences the environment in which trees develop, including light, heat, temperature, humidity, and soil nutrients (Cui et al., 2022). At low stand densities, tree interactions are minimal or weak, rendering niche complementarity effects insignificant. As stand density escalates, interactions intensify, with trees occupying greater space and utilizing more resources (Forrester et al., 2013; Morin, 2015). Initially, NECC increases as trees grow and the stand matures over time. However, as forests evolve from middle-aged to fully mature, there is an increase in the need for nutrients and water to facilitate the outward growth of trees, simultaneously increasing the rates of transpiration.

The outcomes of our study reveal a beneficial association between NECC and increased structural diversity. Previous studies have validated the influence of structural diversity on forest output, which has been successfully included in predictive models for NECC (LaRue et al., 2023). Importantly, an increase in structural diversity amplifies interactions, such as competition among trees. A varied assortment of structures generates openings in the canopy, enhancing light penetration and promoting a vibrant natural habitat. Layered canopy structures, along with diverse layering of trees, frequently occupy greater spatial areas and exploit a broader range of resources, which is a concept that is elucidated by the principle of niche complementarity (Morin, 2015). The complex design of a stand contributes to minimizing temperature fluctuations, enhancing soil moisture retention, facilitating litter decomposition, and promoting nutrient recycling, thereby improving resource utilization efficiency and increasing both NECC and biomass growth (Crockatt and Bebber, 2015; Schwarz et al., 2014). Although cold–temperate forests lack the characteristic stratification found in subtropical forests, their structural diversity is essential for influencing ecosystem services.

### 4.2 Impact of environmental factors on net ecosystem carbon change

Observations reveal that climate variables, specifically MAT and MAP, exert the greatest influence on NECC among environmental factors, with increasing temperatures and precipitation increasing NECC. Temperature fluctuations contribute to clarifying the spatial pattern of NECC (Liu et al., 2014; Ni et al., 2022). Moreover, the efficacy of forests is constrained by water availability, as precipitation governs water distribution, subsequently affecting tree habitats. The findings of our study indicate that geographical factors, particularly elevation, significantly affect NECC. This was due mainly to topographic factors affecting the spatial distributions of solar radiation and precipitation, together with soil moisture, nutrients, and depth, resulting in intricate impacts on NECC within different ecosystems (Wu et al., 2021). Nevertheless, elements such as slope and gradient have been identified as critical drivers influencing NECC in some studies (He et al., 2023; Jafarian et al., 2023). However, within the parameters of our research area, the impact of these components is less pronounced than that of height. This may be attributed to the predominant tree species (*Larix gmelinii* and *Betula platyphylla*) exhibiting markedly low sensitivity to slope orientation and gradient. *Larix gmelinii*, recognized for its strong cold resistance and moderate water requirements, can thrive on both steep and shaded slopes. *Betula platyphylla* necessitates increased moisture; nonetheless, its tolerance to shadow and cold enables it to flourish over diverse slopes and gradients, mitigating the effects of fluctuations in light and temperature.

### 4.3 Beneficial impact of forest age on net ecosystem carbon change via structural diversity and soil factors

The effects of soil and especially structural diversity on NECC increased with stand age, as predicted. These greater effects may be partly due to increasing tree–tree interactions and complementary effects (Wang et al., 2022), coupled with increased build-up of soil organic matter and heightened microbial activity in the soil, which can intensify the impact on NECC. With respect to forest age, the greater positive effects of structural diversity with stand age illustrate that processes such as resource partitioning, facilitation or trophic interactions may result in greater benefits for tree growth in plots with high stand ages than in plots with low stand ages (Hatami et al., 2020; Zhang et al., 2020). This finding is supported by those of previous studies showing that as a forest advances through its developmental phases, its structure becomes increasingly intricate, and the linkages between resource distribution among trees and biodiversity become more evident. Structural variety enhances resource utilization efficiency and stabilizes ecosystem functioning by affecting tree distribution, density, canopy architecture, and interorganism interactions (Aakala et al., 2013; Buechling et al., 2017; Jiang et al., 2018). In addition, as the trees in the forest undergo maturation, organic matter from litterfall and root systems progressively accumulates, leading to an increase in the soil organic matter content. This accumulation not only augments soil fertility but also improves the physical structure of the soil (Post and Kwon, 2000), including the formation and stabilization of soil aggregates, thereby augmenting soil aeration and water retention capacity. Soil structure directly influences root development and nutrient assimilation. A well-developed soil structure facilitates the efficient cycling of water and nutrients, which is imperative for the optimal growth and vitality of trees (Jandl et al., 2007).

### 4.4 Role of forest age in mitigating the impact of climate and topographical variations on net ecosystem carbon change

The results of our study reinforce the mitigating effect of forest age in mitigating climate and topographic shifts, showing that mature forests are less susceptible to climate and topographical changes than their younger counterparts are. Research has indicated that mature forests are more sensitive to climate change than younger forests are (Zhang et al., 2024b). This disagreement may arise from variations in the chosen study regions, as the Da Xing’an Mountains are situated in a cold–temperate humid to semihumid zone. The growth dynamics of several species may be affected by habitat factors. Trees in arid environments are more vulnerable to climate change than those in humid environments are, irrespective of their presence in mature or young forests (Xue et al., 2025). The first piece of evidence proves that there is indeed a dampening effect of forest heterogeneity within the ecosystem on the climate sensitivity of forests, which can be related to the fact that multiage forests benefit from increasing structural complexity (de Wergifosse et al., 2022; Jandl et al., 2019). Compared with young forests, overaged forests exhibit greater diversity in the age structure, characterized by a combination of young, middle-aged, and mature trees. The creation of vertical stratification enhances ecosystem complexity and stability. This stratification provides gradients in biomass (deadwood and living aboveground biomass) and different ways to allocate carbon (Maréchaux et al., 2021). This stratification has also enabled the development of unique carbon distribution tactics and distinguished the ecological roles of plants and animals, thus increasing biodiversity and bolstering the ecosystem’s ability to withstand environmental disruptions (Lafond et al., 2014; Pardos et al., 2021). From a functional perspective, a forest of diverse ages exhibits a variety of physiological and life cycle characteristics among its trees. This variety translates into a greater capacity to absorb and store carbon, thereby mitigating the effects of climate change, even in a mosaic of even-aged patches, as we simulated in this study. Young trees grow rapidly and absorb significant amounts of CO2 from the atmosphere, contributing to carbon capture, and are more efficient in converting photosynthates in biomass (Campioli et al., 2015; Collalti et al., 2014; Viet et al., 2023). In contrast, older trees accumulate more biomass and serve as long-term carbon sinks and regenerative shelters. Concurrently, the increased diversity between age groups offsets the beneficial and detrimental effects linked to each age group. Consequently, in fluctuating weather scenarios, the existence of various age groups in forest communities is vital for preserving their functional variety since this diversity offers advantages in terms of resilience and the ability to adapt to climatic shifts (Ehbrecht et al., 2021; Kauppi et al., 2022; Zampieri et al., 2021). Furthermore, the age of forests plays a role in lessening the impact of topographical alterations. Forest age distribution plays a role in shaping how topography impacts forest ecosystems (Durán Zuazo and Rodríguez Pleguezuelo, 2008; Selkimäki et al., 2012). For example, forests of various ages, owing to their intricate structures, might show greater resilience to topographical changes such as soil erosion and landslides. Such varied structures aid in stabilizing the soil, diminishing erosion, and increasing the forest’s ability to retain water.

## 5 Limitations

The factors influencing NECC were investigated over a relatively short period from 2005 to 2010. An important limitation of this study, which must be acknowledged, is that five years is insufficient to capture the dynamics of slow-growing cold-temperate coniferous forests. Short-term fluctuations caused by extreme climate events, insect outbreaks, or other transient disturbances may disproportionately influence the results. Such events often operate on longer time scales and can profoundly influence forest development pathways; however, short-term data may fail to adequately capture their full effects. Furthermore, although species-specific age classification criteria were applied during data processing, the model was not stratified by individual tree species. It is recognized that significant differences may exist among species in developmental stages—for example, a 40-year-old birch may be considered mature, while a larch of the same age is still young.These differences in growth dynamics and life history strategies could potentially influence the relationships among model variables. Although the current modeling approach aggregated species to maintain sufficient statistical power and model stability, it inherently assumes a certain degree of ecological similarity in stand development across species. Future studies should incorporate extended datasets, integrate assessments of extreme climatic events, and combine species-specific analyses or multi-group structural equation modeling to more accurately evaluate potential impacts on forest ecosystems. Nonetheless, the commonality in stand dynamics and silvicultural practices within the studied forest system provides an ecological rationale for the integrated modeling approach adopted in this study.

## 6 Conclusions

Using NFCI data from 2005 to 2010, PLS–SEM was employed to examine the correlations among stand variables, structural diversity, and environmental variables in assessing the NECC of natural forests. Our research indicates that stand variables are the principal determinants of NECC, with direct influences being notably substantial. Furthermore, the age of forests enhances the impact of structural variety and soil on NECC while alleviating the effects of climate and topography. In light of impending climate change, forest management practices must be customized to various stages of forest development. In young forests during the early successional stage, the application of moderate and selective thinning techniques can diminish competition among trees, foster the establishment of dominant species, and preserve a degree of species diversity to improve structural complexity. In middle-aged and near-aged forests in the mid-successional and mature phases, implementing moderate thinning and selective logging can foster a multilayered vertical structure, optimize light conditions, promote understory regeneration, and increase ecosystem stability. In mature and overaged forests in the late successional stage, excessive intervention should be curtailed while preserving a proportion of standing dead trees and fallen logs to increase soil nutrient cycling and fertility. Furthermore, artificial regeneration can be effectively executed by introducing seedlings of varying ages to improve the age structure variety within a stand. The implementation of these measures enables forests to significantly contribute to climate change mitigation, improve carbon sequestration, and preserve biodiversity. Our research findings establish a foundational theoretical framework for the development of sustainable forest management practices in the Da Xing’an Mountains of Northeast China.

## Data Availability

The raw data supporting the conclusions of this article will be made available by the authors, without undue reservation.
