# Supplementary material for: Factors influencing net ecosystem carbon change in cold-temperate coniferous forests of the Da Xing’an Mountains: analysis across developmental stages based on stand, structural, and environmental factors
Source: Front Plant Sci. 2025 Nov 24;16:1663271. doi: 10.3389/fpls.2025.1663271 (PMC12683666; doi:10.3389/fpls.2025.1663271)
Supplement: Supplementary file 1 [file Table1.docx]

The variance inflation factor (VIF) of the independent variables

| Climate | | Stand | | Soil | | Structure diversity | | Topography | |
| --- | --- | --- | --- | --- | --- | --- | --- | --- | --- |
| Variables | VIF | Variables | VIF | Variables | VIF | Variables | VIF | Variables | VIF |
| MAT | 1.44 | Age | 1.82 | DEPTH | 1.18 | ISCD | 1.06 | Altitude | 3.25 |
| MWMT | 326 | Dg | 3.12 | REF_BULK_DENSITY | 3.52 |  |  | Aspect | 1.09 |
| MCMT | 811 | Density | 2.06 | OC | 2.32 |  |  | Slope | 1.29 |
| TD | 471 |  |  | PH_H2O | 12.66 |  |  |  |  |
| MAP | 1.14 |  |  | CEC_SOIL | 2.81 |  |  |  |  |
| AHM | 29.46 |  |  | BS | 4.98 |  |  |  |  |
| DD_0 | 201 |  |  | TEB | 3.01 |  |  |  |  |
| DD5 | 1062 |  |  | CACO3 | 5.91 |  |  |  |  |
| DD18 | 111 |  |  | ESP | 1.67 |  |  |  |  |
| NFFD | 264 |  |  | ECE | 2.10 |  |  |  |  |
| PAS | 12.72 |  |  |  |  |  |  |  |  |
| EMT | 64.73 |  |  |  |  |  |  |  |  |
| EXT | 68.50 |  |  |  |  |  |  |  |  |
| EREF | 15.22 |  |  |  |  |  |  |  |  |
| CMD | 12.24 |  |  |  |  |  |  |  |  |
| RH | 39.98 |  |  |  |  |  |  |  |  |

The variance Pearson of the independent variables

| Climate | | Stand | | Soil | | Structure diversity | | Topography | |
| --- | --- | --- | --- | --- | --- | --- | --- | --- | --- |
| Variables | Pearson | Variables | Pearson | Variables | Pearson | Variables | Pearson | Variables | Pearson |
| MAT | .316** | Age | .283** | DEPTH | 0.251** | ISCD | -.225** | Altitude | -.221** |
| MWMT | .316** | Dg | .258** | REF_BULK_DENSITY | -0.02 |  |  | Aspect | 0.026 |
| MCMT | .189** | Density | .362** | OC | -0.013 |  |  | Slope | -.158** |
| TD | 0 |  |  | PH_H2O | -0.049 |  |  |  |  |
| MAP | .273** |  |  | CEC_SOIL | -0.01 |  |  |  |  |
| AHM | .304** |  |  | BS | -0.027 |  |  |  |  |
| DD_0 | -.246** |  |  | TEB | -0.012 |  |  |  |  |
| DD5 | .308** |  |  | CACO3 | -0.042 |  |  |  |  |
| DD18 | -.272** |  |  | ESP | 0.02 |  |  |  |  |
| NFFD | .288** |  |  | ECE | 0.021 |  |  |  |  |
| PAS | .292** |  |  |  |  |  |  |  |  |
| EMT | -.122** |  |  |  |  |  |  |  |  |
| EXT | .199** |  |  |  |  |  |  |  |  |
| EREF | .320** |  |  |  |  |  |  |  |  |
| CMD | .187** |  |  |  |  |  |  |  |  |
| RH | .245** |  |  |  |  |  |  |  |  |

The variance RF of the independent variables

| Climate | | Stand | | Soil | | Structure diversity | | Topography | |
| --- | --- | --- | --- | --- | --- | --- | --- | --- | --- |
| Variables | RF | Variables | RF | Variables | RF | Variables | RF | Variables | RF |
| MAT | 23.08** | Age | 22.71** | DEPTH | 23.27** | ISCD | 8.47* | Altitude | 8.59* |
| MWMT | 6.50 | Dg | 23.01** | REF_BULK_DENSITY | -0.45 |  |  | Aspect | 2.46 |
| MCMT | 10.53 | Density | 23.27** | OC | -0.77 |  |  | Slope | 4.37 |
| TD | 12.15 |  |  | PH_H2O | 0.68 |  |  |  |  |
| MAP | 32.22** |  |  | CEC_SOIL | 0.21 |  |  |  |  |
| AHM | 7.65 |  |  | BS | 1.41 |  |  |  |  |
| DD_0 | 9.98 |  |  | TEB | 0.58 |  |  |  |  |
| DD5 | 8.01 |  |  | CACO3 | 2.74 |  |  |  |  |
| DD18 | 7.52 |  |  | ESP | 0.18 |  |  |  |  |
| NFFD | 5.69 |  |  | ECE | -0.68 |  |  |  |  |
| PAS | 17.88 |  |  |  |  |  |  |  |  |
| EMT | 7.95 |  |  |  |  |  |  |  |  |
| EXT | 7.53 |  |  |  |  |  |  |  |  |
| EREF | 3.96 |  |  |  |  |  |  |  |  |
| CMD | 6.69 |  |  |  |  |  |  |  |  |
| RH | 4.95 |  |  |  |  |  |  |  |  |
